# Prevalence and Risk Factors of Iron Deficiency and Iron Deficiency Anemia in Preschool Children of China: A Prospective Observational Study

**DOI:** 10.1002/fsn3.71110

**Published:** 2025-10-15

**Authors:** Changjuan Gu, Zengcheng Wang, Huijun Zhao, Xiaotian Xie

**Affiliations:** ^1^ Department of Pediatrics Tongji Hospital, School of Medicine, Tongji University Shanghai People's Republic of China; ^2^ Department of Pediatrics Suzhou Kowloon HospitalShanghai Jiaotong University, School of Medicine Suzhou People's Republic of China

**Keywords:** anemia, breastfeeding, children, iron deficiency, nutrition

## Abstract

To explore the prevalence and risk factors of iron deficiency (ID) and iron deficiency anemia (IDA) in preschool children. A total of 3503 preschool children (3–4 years) who have normal physical checkups for kindergarten admission have been studied. The prevalence of children's ID and IDA has been detected, and the main influencing factors of children's ID and IDA were analyzed by factor comparison and logistic regression. The prevalence of ID and IDA was 25.4% and 7.5%, respectively, in the total of 3503 children. Male gender, exclusive breastfeeding, delay of solid food supplement, picky eating, recent respiratory infection and diarrhea, and parents without a bachelor's degree or above or other places' household registration are associated with children's ID and IDA. Among these, male gender, exclusive breastfeeding, not adding food in time (such as > 6 months after birth), and picky eating are the independent risk factors for children's ID and IDA. The prevalence of children's ID and IDA is still at a high level. Among 3–4 years preschool children, boys are at a higher risk of developing ID and IDA. Feeding‐related factors, including exclusive breastfeeding and picky eating habits, are particularly important for the occurrence of ID. Consequently, it is necessary to conduct further investigations and research on different age groups in various regions.

## Graphical abstract

The prevalence of ID and IDA among preschool children are still high in China, even in a relatively developed region. The prevalence rate of ID exceeds that of IDA by almost threefold, showing the underrecognized burden of ID and emphasizing the urgency of early screening and prevention strategies. Male gender, exclusive breastfeeding beyond six months, delayed introduction of complementary foods, and picky eating identified are independent risk factors of ID and IDA.

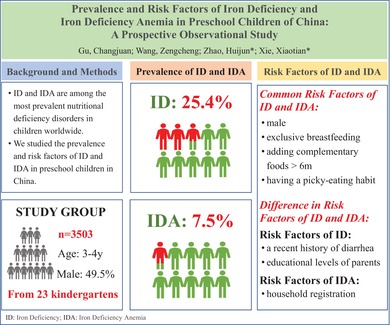

## 1 Introduction

Iron deficiency anemia (IDA) and iron deficiency (ID) are among the most prevalent nutritional deficiency disorders in children worldwide (Leung et al. 2024). IDA, in particular, is characterized by a reduction in hemoglobin levels due to insufficient iron availability, while ID represents an earlier stage where iron stores are depleted, yet anemia may not be fully manifested (Camaschella 2019). These conditions are not only common but also pose significant threats to children's health.

Recent research (Pivina et al. 2019) has also highlighted the critical impact of ID during the early stages of life, especially in infants. The brain is in a rapid development phase during infancy, and iron is essential for normal brain development. ID during this period may lead to irreversible damage to brain development, potentially affecting cognitive function, learning ability, and behavior in the long term.

Over the past few decades, there has been an increase in the understanding of anemia and the implementation of intervention measures for this condition. In China, due to its vast territory and diverse regional characteristics, even the Chinese government has made reducing children's IDA a key goal; nevertheless, the incidence of children's IDA in various regions remains at a relatively high level (Cheung et al. 2023; Zheng et al. 2020). In particular, in economic development zones with large‐scale population inflows, a high proportion of child‐bearing‐age populations, and significant differences in the educational levels of parents, relevant epidemiological studies are urgently needed. Moreover, before the effective implementation of national intervention measures and programs, it is essential to assess the prevalence and severity of ID and IDA and their potential risk factors.

Therefore, this study is to explore the prevalence and risk factors of ID and IDA in preschool children and provide a scientific basis for effective prevention and targeted treatment, aiming at improving the overall health level of children.

## 2 Materials and Methods

### 2.1 Study Design

The data for this study were collected from preschool–age children undergoing kindergarten admission physical examinations in May 2023. This research project was a collaborative effort between the education and health departments of Suzhou Industrial Park and the Municipal Science and Technology Bureau. It was led by the Department of Pediatrics of Tongji Hospital of Tongji University and Suzhou Kowloon Hospital of Shanghai Jiaotong University, with the assistance of relevant kindergartens and nurseries. The study followed the Strengthening the Reporting of Observational Studies in Epidemiology (STROBE) statement.

### 2.2 Diagnostic Criteria

#### 2.2.1 Diagnostic Criteria for IDA

The diagnostic criteria for IDA were based on the following:

- Hemoglobin (HB) reduction and meet the anemia diagnostic criteria for children.
- Microcytic hypochromic characteristics: Mean corpuscular volume (MCV) < 80 fL, mean corpuscular hemoglobin (MCH) < 27 pg, and mean corpuscular hemoglobin concentration (MCHC) < 310 g/L.
- Related factors that could lead to ID.
- In the iron metabolism test, at least two of the following four items were present: Serum ferritin (SF) < 15 μg/L, serum iron (SI) < 10.7 μmol/L, total iron‐binding capacity (TIBC) > 62.7 μmol/L, and transferrin saturation (TS) < 15%.
- Effective response to iron therapy: After 4 weeks of iron therapy, Hb increased by more than 20 g/L.
- Bone marrow examination: The extracellular iron was significantly reduced, and the sideroblasts < 15%.
- Differential diagnosis: Excluding other diseases that could present with microcytic hypochromic anemia.

Those who met the first and second items above and excluded other diseases with microcytic hypochromic anemia could be tentatively diagnosed with IDA.

#### 2.2.2 Diagnostic Criteria for ID

The diagnostic criteria for ID were as follows:

- Factors that could lead to ID
- SF was below the normal range
- Microcytic hypochromic characteristics
- Hb had not yet reached the degree of anemia.

### 2.3 Patients

#### 2.3.1 Inclusion Criteria

- Preschool children aged between 3 and 4 years old.
- Permission from parents: The parents and children were willing to cooperate with the examination, complete the questionnaire survey, and sign the informed consent.

#### 2.3.2 Exclusion Criteria

- History of blood diseases, congenital malformations, or family history of genetic diseases.
- The physical examination showed special facial features, jaundice, splenomegaly, or other physical signs related to blood diseases.
- There was a history of diseases or recent surgeries during the detection period.

### 2.4 Detection Items

#### 2.4.1 Physical Examination

The physical examination for preschool children included routine items for kindergarten admission. This involved a comprehensive assessment of physical development, such as measuring height, weight, and head circumference to evaluate the growth status of children. Behavioral ability was also examined, observing the children's motor skills, language development, and social interaction abilities. Clinical physical examinations of the skin and mucosa were carried out to check for any signs of skin diseases, jaundice, or other abnormal conditions. The head, face, five sense organs, as well as the thoracic and abdominal internal organs, were carefully examined. For example, auscultation of the heart and lungs was performed to detect any abnormal heart sounds or respiratory problems, and palpation of the abdomen was done to check for the presence of an enlarged liver, spleen, or other abdominal masses.

#### 2.4.2 Laboratory Tests

- **Blood Routine Test**: The dodecyl sulfonate method was used (standard instrument: SYSMEX SN‐A3430). This test detected routine items such as the counts of red blood cells (RBC), white blood cells (WBC), and platelets in the peripheral blood, HB value, and RBC morphological parameters (MCV, MCH, and MCHC).
- **SF**: The chemiluminescent microparticle immunoassay was adopted (standard instrument: ARCHITECT i2000SR). SF is an important indicator reflecting the body's iron storage status. By measuring SF levels, it is possible to determine whether a child has ID at an early stage.
- **SI**: The ferrozine method was employed (standard instrument: ARCHITECT c16000 system). Measuring SI can help understand the level of iron in the blood that is available for physiological functions, which is crucial for diagnosing iron‐related disorders.

#### 2.4.3 Questionnaire Survey

A questionnaire survey was designed and completed with the close cooperation of kindergartens and nurseries based on the epidemiological characteristics and possible influencing factors of children's IDA suggested by previous studies.

- **Birth History**: Information such as gestational age (full‐term or premature), number of fetuses (single fetus or multiple fetuses), birth weight, and average gestational age was collected.
- **Feeding History**: The feeding methods within 6 months after birth were investigated, including exclusive breastfeeding, mixed feeding with added formula milk or relying solely on formula milk, and artificial feeding. The time of complementary food addition was also recorded. Additionally, with the assistance of kindergartens and nurseries, it was determined whether there were obvious picky eating habits.
- **Common Disease History**: Information on whether there was a clear history of respiratory tract infections or diarrhea within the recent 6 months, and the number of illnesses was collected.
- **Primary Caregiver Information**: This included details about the composition of the main primary caregiver in the family (whether grandparents were involved), the ages, educational levels, and household registration locations of the parents.

Based on the test results, the children under examination were divided into a normal group, an ID group, and an IDA group. The data from the questionnaire were then used to statistically compare and analyze the main influencing factors leading to the occurrence of ID and IDA in local children.

### 2.5 Statistical Analysis

Data analysis was performed using SPSS 20.0 statistical software. For count data, they were expressed as frequency (*n*) and rate (%). The chi‐square test was used for comparisons between groups. For measurement data, they were expressed as mean ± standard deviation (^−^x ± s), and the t‐test was used for comparisons between groups. Logistic regression analysis was employed to identify independent risk factors. A value of *p* < 0.05 was considered statistically significant.

## 3 Results

**FIGURE 1:**
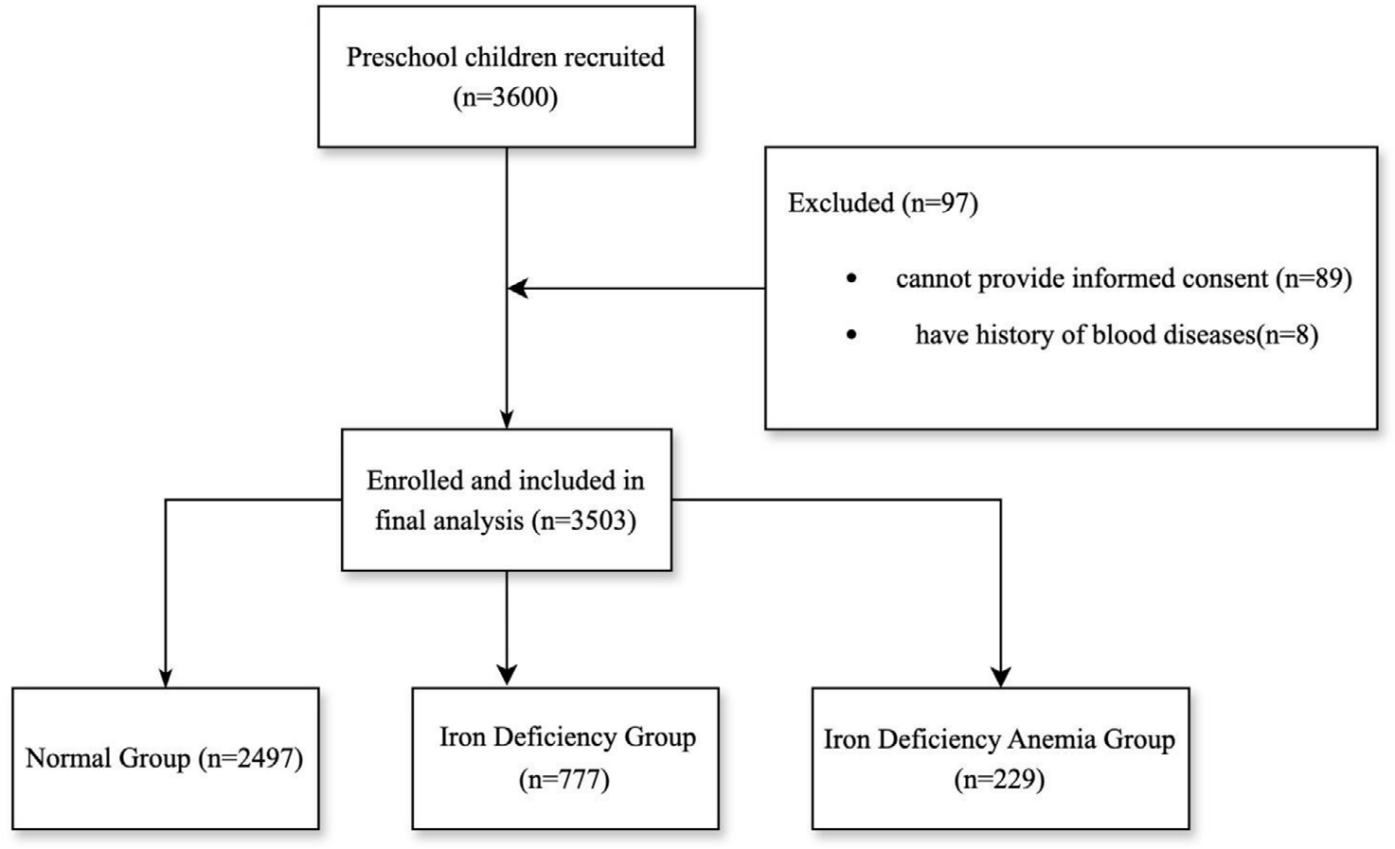
The study flow diagram.

A total of 3600 children, aged between 3 and 4 years old, from 23 kindergartens (7 public and 16 private) were assessed for eligibility for the study. 97 children were excluded because they could not provide informed consent or had a history of blood diseases. Finally, 3503 children were enrolled in the study (Figure 1). Among them, 1734 were boys and 1769 were girls (Table 1).

**TABLE 1:** Factors influencing the incidence of ID and IDA in children (univariate analysis).

Variables		Total number (3503)	Normal group (%)	ID group (%)	X2	*p*	IDA group (%)	X2	*p*
Gender	Male	1734	1159 (66.8)	445 (25.7)	27.946	0.000	130 (7.5)	9.02	0.003
	Female	1769	1338 (75.6)	332 (18.8)			99 (5.6)		
Number of fetuses	Multiple fetuses	43	26 (60.5)	13 (30.2)	2.01	0.156	4 (9.3)	0.421	0.517
	Single fetus	3460	2471 (71.4)	764 (22.1)			225 (6.5)		
Gestation age	Full‐term	3346	2389 (71.4)	741 (22.1)	0.134	0.715	216 (6.5)	0.903	0.342
	Premature	157	108 (68.8)	36 (22.9)			13 (8.3)		
Birth weight	< 2.5 kg	85	56 (65.9)	21 (24.7)	1.602	0.449	8 (9.4)	1.647	0.439
	2.5–4 kg	3117	2221 (71.3)	697 (22.4)			199 (6.4)		
	> 4.0 kg	301	220 (73.1)	59 (19.6)			22 (7.3)		
Feeding method	Exclusive breastfeeding	2029	1293 (63.7)	580 (28.6)	131.615	0.000	156 (7.7)	22.538	0.000
	Artificial feeding	211	161 (76.3)	41 (19.4)			9 (4.3)		
	Mixed feeding	1263	1043 (82.6)	156 (12.4)			64 (5.1)		
Complementary foods	≤ 6 months	2981	2202 (73.9)	597 (20.0)	61.568	0.000	182 (6.1)	14.503	0.000
	> 6 months	522	295 (56.5)	180 (34.5)			47 (9.0)		
Picky eating	Yes	634	323 (50.9)	238 (37.5)	130.681	0.000	73 (11.5)	60.616	0.000
	No	2869	2174 (75.8)	539 (18.8)			156 (5.4)		
Respiratory infection*	Yes	2583	1825 (70.7)	601 (23.3)	5.606	0.018	157 (6.1)	2.168	0.141
	No	920	672 (73.0)	176 (19.1)			72 (7.8)		
Respiratory infection frequency	≤ 2 times	2203	1588 (72.1)	468 (21.2)	2.872	0.090	147 (6.7)	0.032	0.858
	> 2 times	1300	909 (69.9)	309 (23.8)			82 (6.3)		
Diarrhea*	Yes	2433	1716 (70.5)	556 (22.9)	2.242	0.134	161 (6.6)	0.245	0.620
	No	1070	781 (73.0)	221 (20.7)			68 (6.4)		
Diarrhea frequency	≤ 2 times	3013	2174 (72.2)	643 (21.3)	9.167	0.002	196 (6.5)	0.402	0.526
	> 2 times	490	323 (65.9)	134 (27.3)			33 (6.7)		
Caregiver person	Parents	1969	1403 (71.3)	440 (22.3)	0.251	0.882	126 (6.4)	0.489	0.783
	Co‐caregivers	1534	1094 (71.3)	337 (22.0)			103 (6.7)		
Mother age	≤ 32 years old	1741	1221 (70.1)	395 (22.7)	0.89	0.345	125 (7.2)	2.714	0.099
	> 32 years old	1762	1276 (72.4)	382 (21.7)			104 (5.9)		
Mother education	Below Bachelor's Degree	1148	755 (65.8)	314 (27.4)	27.903	0.000	79 (6.9)	1.794	0.180
	Bachelor's Degree or Above	2355	1742 (74.0)	463 (19.7)			150 (6.4)		
Mother household registration	Local	2333	1699 (72.8)	501 (21.5)	3.413	0.065	133 (5.7)	9.447	0.002
	Nonlocal	1170	798 (68.2)	276 (23.6)			96 (8.2)		
Father age	≤ 32 years old	1273	887 (69.7)	293 (23.0)	1.229	0.268	93 (7.3)	2.359	0.125
	> 32 years old	2230	1610 (72.2)	484 (21.7)			136 (6.1)		
Father education	Below bachelor's degree	879	556 (63.3)	259 (29.5)	38.818	0.000	64 (7.3)	3.853	0.050
	Bachelor's degree or above	2624	1941 (74.0)	518 (19.7)			165 (6.3)		
Father registration	Local	2237	1617 (72.3)	481 (21.5)	2.096	0.148	139 (6.2)	1.508	0.219
	Non‐local	1266	880 (69.5)	296 (23.4)			90 (7.1)		

* Respiratory infection and diarrhea occurred in the recent 6 months.

According to the diagnostic criteria, 777 cases (25.4%) of ID and 229 cases (7.5%) of IDA were detected, respectively. Statistical comparison showed that the prevalence rates of ID and IDA in boys were significantly higher than those in girls. The specific data are shown in Table 1.

The univariate analysis showed factors such as gestational age, number of fetuses, and birth weight had no significant differences between the normal group and ID/IDA group. The prevalence of ID and IDA in children, who were exclusively breastfed, did not add complementary foods in time, or had obvious picky eating habits, was significantly increased. For children with a history of respiratory tract infections or diarrhea within the recent 6 months, the prevalence of ID was notably elevated. Those whose fathers or mothers did not obtain higher education diplomas and whose mothers had non‐local household registrations demonstrated a high incidence of both ID and IDA. In contrast, neither the participation of grandparents in the upbringing process nor the parental age exhibited any correlation with the prevalence of ID and IDA. The specific data are shown in Table 1.

Based on the results of the univariate analysis, Logistic regression analysis was carried out. The results showed that male gender, exclusive breastfeeding, not adding complementary foods in time (more than 6 months after birth), and having a picky eating habit were all independent risk factors for the incidence of ID and IDA in preschool children.

**FIGURE 2:**
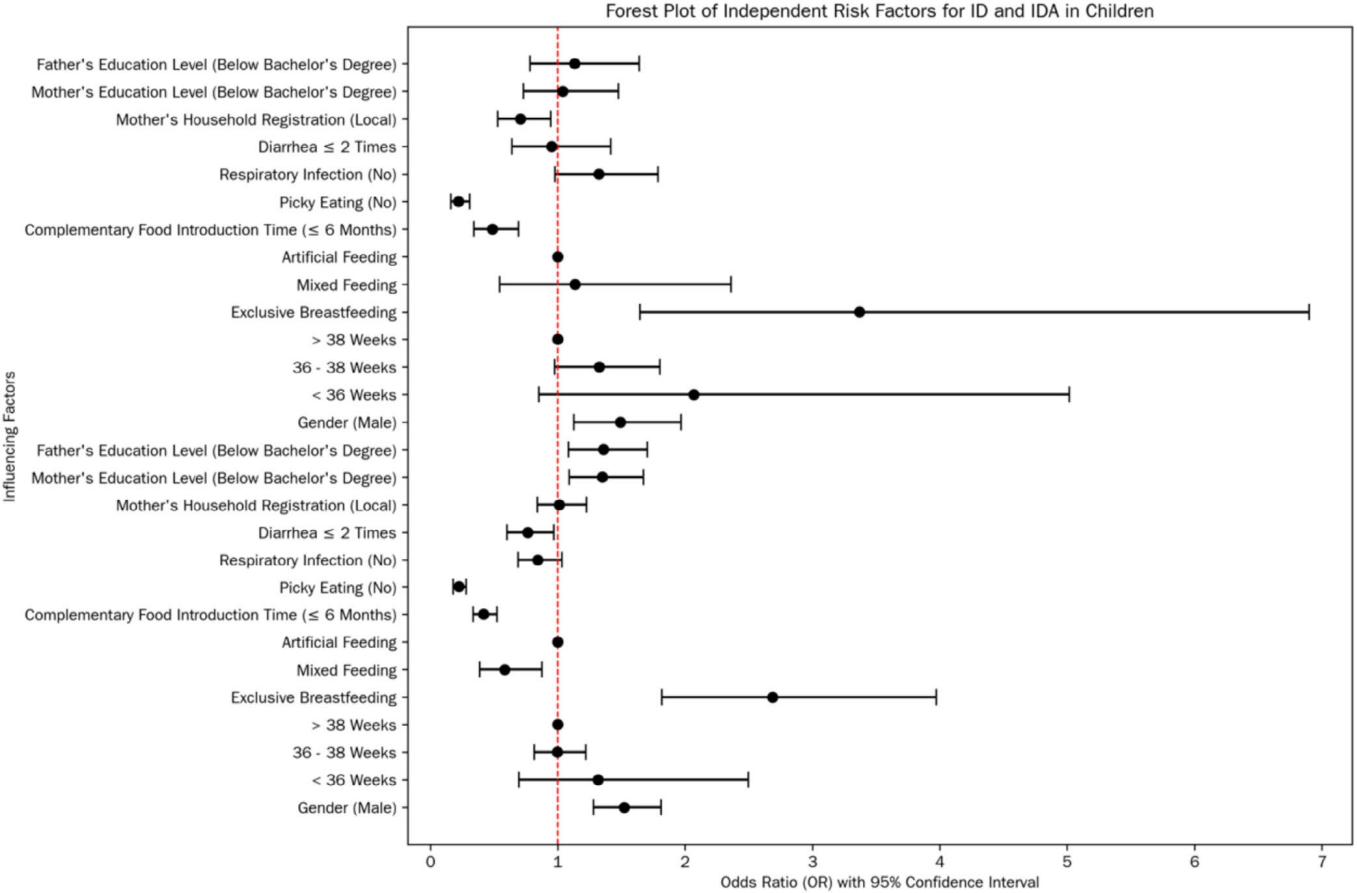
Forest plot of independent risk factors for ID and IDA in children.

In addition, there were some differences in independent risk factors between ID and IDA. A recent history of diarrhea, the educational levels of parents were independent risk factors for the incidence of ID, while household registration was an independent risk factor for the incidence of IDA. Premature birth and a recent history of respiratory tract infections were not independent risk factors for ID and IDA. The specific data are shown in Table 2 and Figure 2.

**TABLE 2:** Independent risk factors for ID and IDA in children (multifactorial analysis).

Influencing factors	B	SE	OR	95% CI	*p*
Influencing factors of ID group					
Gender (male)	0.420	0.089	1.522	1.279–1.811	0.000
Gestational week					
< 36 weeks	0.275	0.326	1.317	0.695–2.496	0.399
36–38 weeks	−0.003	0.103	0.997	0.815–1.220	0.979
> 38 weeks	—	—	1	—	—
Feeding method					
Exclusive breastfeeding	0.988	0.199	2.686	1.817–3.971	0.000
Mixed feeding	−0.541	0.209	0.582	0.387–0.876	0.009
Artificial feeding	—	—	1	—	—
Complementary food introduction time (≤ 6 months)	−0.874	0.114	0.417	0.334–0.522	0.000
Picky eating (no)	−1.496	0.113	0.224	0.179–0.280	0.000
Respiratory infection* (no)	−0.170	0.103	0.843	0.689–1.033	0.099
Diarrhea* ≤ 2 times	−0.271	0.122	0.763	0.601–0.969	0.026
Mother's household registration (local)	0.013	0.097	1.013	0.839–1.225	0.890
Mother's education level (below bachelor's degree)	0.301	0.109	1.351	1.090–1.674	0.006
Father's education level (below bachelor's degree)	0.307	0.115	1.359	1.085–1.704	0.008
Influencing factors of IDA group					
Gender (male)	0.398	0.142	1.49	1.127–1.969	0.005
Gestational week					
< 36 weeks	0.726	0.453	2.067	0.851–5.018	0.109
36–38 weeks	0.282	0.156	1.326	0.976–1.801	0.071
> 38 weeks	—	—	1	—	—
Feeding method					
Exclusive breastfeeding	1.215	0.366	3.369	1.645–6.901	0.001
Mixed feeding	0.127	0.374	1.135	0.545–2.362	0.735
Artificial feeding	—	—	1	—	—
Complementary food introduction time (≤ 6 months)	−0.720	0.180	0.487	0.342–0.692	0.000
Picky eating (no)	−1.506	0.168	0.222	0.160–0.308	0.000
Respiratory infection (no)	0.280	0.154	1.323	0.979–1.788	0.069
Diarrhea ≤ 2 times	−0.050	0.203	0.952	0.639–1.417	0.807
Mother's household registration (local)	−0.347	0.149	0.707	0.528–0.946	0.020
Mother's education level (below bachelor's degree)	0.038	0.179	1.039	0.732–1.475	0.832
Father's education level (below bachelor's degree)	0.123	0.189	1.131	0.781–1.639	0.514

* Respiratory infection and diarrhea occurred in the past 6 months.

## 4 Discussion

ID and IDA are significant global health issues, particularly affecting children and pregnant women (Camaschella 2019). The prevalence is high in regions with low Socio‐Demographic Indexes, such as sub‐Saharan Africa and South Asia (Camaschella 2019; Liu et al. 2024). The samples of this study were from Suzhou Industrial Park in China, which is a national‐level economic development zone established under an international cooperation model. The results showed that the incidence of IDA and ID in this group of children still reached 25.4% and 7.5%, respectively. This indicates that even in the relatively developed areas in terms of economic and cultural levels, the overall popularization degree of knowledge related to children's iron metabolism is still lagging behind.

ID is a precursor to IDA and is more prevalent than IDA, especially in children (Turawa et al. 2021; Gupta et al. 2016). ID in young children, even without anemia, can lead to significant neurocognitive and developmental challenges, including cognitive impairments, psychomotor delays, and behavioral issues [3.9]. Our research findings indicate that the prevalence rate of ID exceeds that of IDA by almost threefold. Paradoxically, clinicians tend to allocate less attention to ID. In the case of young children, whose brains are at a critical stage of development, the detrimental effects induced by ID frequently prove to be persistent and, consequently, demand heightened attention from all concerned parties.

Among adults, females are more likely to develop IDA due to factors such as menstrual blood loss, pregnancy, and dietary deficiencies (Sedlander et al. 2021; Levi 2019). Nevertheless, when it comes to the pediatric population, the gender‐based variation in the prevalence of IDA remains inconclusive. One study in Sweden and Honduras found that male infants had significantly lower HB levels and other iron status indicators compared to females, with boys having a 10‐fold higher risk of developing IDA by 9 months of age (Domellöf et al. 2002). Conversely, other research indicates a higher prevalence of IDA among females, particularly in adolescents, with rates reported as 26% in the Gaza Strip, Palestine (Jalambo et al. 2018), 21.1% in Jatinangor, Indonesia (Sari et al. 2022), and 40% in India (Scott et al. 2022).

In our study, male gender was identified as a clear risk factor for ID and IDA in 3–4 years old preschool children. It might be due to rapid weight gain, larger blood volume, and potentially higher androgen levels, which may influence iron metabolism and requirements. Some studies suggest that boys may be more susceptible to the cognitive impacts of ID (Farhan et al. 2024). The higher incidence of cognitive impairment in boys may be associated with a higher risk of early ID. Our results reflected the importance of early detection and intervention, and further research is needed to fully understand the gender‐specific impacts and to develop effective prevention strategies.

Exclusive breastfeeding is generally recommended for the first 6 months of life due to its numerous health benefits. However, our findings and several previous studies indicate that exclusive breastfeeding beyond 6 months can increase the risk of ID and IDA in infants. This is because breast milk alone may not provide sufficient iron to meet the growing needs of infants after this period (Clark et al. 2017).

Our study demonstrates that a history of poor feeding during infancy (failure to add complementary foods after 6 months) is an independent risk factor for the incidence of ID and IDA in preschool children, which further demonstrates the importance of timely addition of complementary foods. While exclusive breastfeeding is beneficial in the early months, it may increase the risk of ID and anemia if continued beyond 6 months without appropriate dietary interventions.

For preschoolers aged between 3 and 4, another prominent feeding concern pertains to picky eating habits and dietary aversions. Picky eating often results in a limited diet, which can lead to low intakes of iron and zinc, particularly due to reduced consumption of meat, fruits, and vegetables (Taylor and Emmett 2018). Our research reaffirms that children with finicky eating behaviors are more predisposed to developing ID and IDA. Ensuring a balanced diet rich in iron and other essential nutrients is crucial for preventing ID and anemia in children.

Diarrhea can lead to nutrient malabsorption, including iron, particularly in young children (Chandyo et al. 2015). Our research findings suggest that persistent diarrhea (> 2 times in recently 6 months) is a risk factor for ID, but not for IDA. It is shown that while diarrhea can increase the risk of ID, the association with IDA is not significant, suggesting other factors might be involved in the development of anemia. The relationship between diarrhea and IDA is complex, might involve multiple nutritional and environmental factors, which need further exploration.

Similar to previous studies (Al‐Suhiemat et al. 2020), our research found that parental education plays a significant role in influencing the risk of ID and IDA in children. Children with more educated parents are less likely to develop ID and anemia due to higher socioeconomic status and better dietary practices, including a greater understanding of the importance of iron‐rich foods and the role of dietary diversity, which contribute to higher HB levels in children.

Thus, conducting educational programs for parents is of the utmost necessity. These programs can effectively enhance their knowledge about IDA, thereby reducing the prevalence of this condition among children.

Premature birth, being a twin, and low birth weight are recognized as risk factors for ID and IDA in many previous studies (Sundararajan and Rabe 2021; Wirth et al. 2022). However, our research findings present distinctive outcomes; these all are not risk factors for ID or IDA in children after 3 years old. Under unfavorable congenital conditions like premature birth, provided that adequate attention of iron‐supplemented diet promptly, it may not have an impact during early childhood.

Our study has several limitations. For example, there is a limited number of cases and a relatively narrow age distribution. However, the sample selection from China can offer valuable references for similar regions with high development levels and large inflow populations.

## 5 Conclusion

Our study highlights the substantial prevalence of ID (25.4%) and IDA (7.5%) among preschool children in China. Among 3–4 years preschool children, boys are at higher risk of developing ID and IDA. Exclusive breastfeeding beyond 6 months without timely introduction of complementary foods, picky eating habits, and lower parental education levels are independent risk factors. These findings provide actionable insights for targeted interventions.

## Author Contributions

**Changjuan Gu:** conceptualization (equal), data curation (equal), formal analysis (lead), investigation (equal), methodology (equal), resources (equal), validation (equal), visualization (equal), writing – original draft (equal), writing – review and editing (equal). **Zengcheng Wang:** conceptualization (equal), data curation (equal), formal analysis (equal), funding acquisition (lead), investigation (equal), project administration (lead), validation (equal), writing – original draft (equal). **Huijun Zhao:** conceptualization (equal), data curation (equal), methodology (equal), project administration (supporting), supervision (equal), writing – review and editing (equal). **Xiaotian Xie:** conceptualization (equal), investigation (supporting), methodology (lead), project administration (supporting), supervision (equal), writing – review and editing (equal).

## Ethics Statement

This study was in accordance with the Declaration of Helsinki and approved by the Medical Ethics Committee of Suzhou Kowloon Hospital (XJ‐2016‐007).

## Consent

All the parents of children signed the informed consent forms.

## Conflicts of Interest

The authors declare no conflicts of interest.

## Data Availability

Complete data are available from the corresponding author. Deidentified data can be shared upon reasonable request. A request for data sharing and collaboration should be sent to xtxie@163.com.
